# A systematic review protocol to evaluate the psychometric properties of measures of function within adult neuro-rehabilitation

**DOI:** 10.1186/s13643-015-0076-5

**Published:** 2015-06-13

**Authors:** Shannon Pike, Natasha Anne Lannin, Anne Cusick, Kylie Wales, Lynne Turner-Stokes, Stephen Ashford

**Affiliations:** Department of Occupational Therapy, La Trobe University, Melbourne, Australia; Wagga Wagga Ambulatory Rehabilitation Service, Wagga Wagga Health Service, Wagga Wagga, Australia; Occupational Therapy Department, Alfred Health, Melbourne, Australia; School of Health and Society, University of Wollongong, Wollongong, Australia; Illawarra Health and Medical Research Institute, University of Wollongong, Wollongong, Australia; Ageing Work and Health Research Unit, The University of Sydney, Sydney, Australia; The Centre for Excellence in Population Ageing Research, The University of Sydney, Sydney, Australia; Regional Rehabilitation Unit, Northwick Park Hospital, London, UK; Department of Palliative Care, Policy and Rehabilitation, King’s College London, School of Medicine, London, UK

**Keywords:** Botulinum toxin, Upper limb, Activity performance, Participation, Neuro-rehabilitation, Outcome measurement

## Abstract

**Background:**

Spasticity in the upper limb is common after acquired brain impairment and may have a significant impact on the ability to perform meaningful daily activities. Traditionally, outcome measurement in spasticity rehabilitation has focused on impairment, however, improvements in impairments do not necessarily translate to improvements in an individual’s ability to perform activities or engage in life roles. There is an increasing need for outcome measures that capture change in activity performance and life participation.

**Methods/Design:**

We will conduct a systematic review of the psychometric properties of instruments used to measure upper limb functional outcomes (activity performance and participation) in patients with spasticity. Assessments (*n* = 27) will be identified from a recently published systematic review of assessments that measure upper limb function in neurological rehabilitation for adults with focal spasticity, and a systematic review of each assessment will then be conducted. The databases MEDLINE, CINAHL and EMBASE will be searched from inception. Search strategies will include the name of the assessment and the COnsensus-based Standards for the selection of health status Measurement INstruments (COSMIN) published search strategy for identifying studies of measurement properties. The methodological rigour of the testing of the psychometric quality of instruments will be undertaken using the Consensus-based Standards for the Selection of Health Measurement Instruments (COSMIN) checklist. International Classification of Functioning, Disability and Health (ICF) definitions of impairment, activity and participation will be used for content analysis of items to determine the extent to which assessments are valid measures of activity performance and life participation. We will present a narrative synthesis on the psychometric properties and utility of all instruments and make recommendations for assessment selection in practice.

**Discussion:**

This systematic review will present a narrative synthesis on the psychometric properties and utility of assessments used to evaluate function in adults with upper limb focal spasticity. Recommendations for assessment selection in practice will be made which will aid clinicians, managers and funding bodies to select an instrument fit for purpose. Importantly, appropriate assessment selection will provide a mechanism for capturing how applicable to everyday life the outcomes from individualised rehabilitation programs for the upper limb really are.

**Systematic review registration:**

PROSPERO CRD42014013190

**Electronic supplementary material:**

The online version of this article (doi:10.1186/s13643-015-0076-5) contains supplementary material, which is available to authorized users.

## Background

Spasticity is most commonly accepted as being a velocity-dependent increase in stretch reflex with exaggerated tendon jerks that occurs as one part of the upper motor neurone syndrome [1]. Pandyan et al [2] more recently redefined spasticity as disordered sensorimotor control, resulting from an upper motor neurone lesion, presenting as intermittent or sustained involuntary activation of muscles. In recent years, there has been a recognition within neuro-rehabilitation that spasticity management programs must go well beyond treatment of impairments, in line with contemporary understandings of health emerging from the World Health Organisation’s International Classification of Functioning, Disability and Health Framework (ICF) [3]. This framework starts with the assumption that health is not a state independent of individuals in the context of everyday life; thus spasticity, a neuro-muscular condition, cannot be considered independent of the person who has it and their daily life. This makes understanding, measuring and monitoring the impact of neuro-rehabilitation programs on function in everyday life as important as measuring and monitoring spasticity.

Contemporary neuro-rehabilitation uses the ICF as an organising framework. Apart from the environment, there are three domains in the classification—impairment, activity and participation [3]. The ICF defines “impairment” as a problem in body (physiological and psychological) function or structure (anatomical parts of the body), “activity” is the execution of a task or action by an individual and “participation” is involvement in a life situation [3]. In upper limb rehabilitation, function is an important and debated term—because impairments can affect function and it is through function that activity and participation goals can be achieved. The term function is used variably within the literature; it alludes to impairments, activity performance and/or participation in life situations in addition to associations with active task performance. Whilst concepts associated with “function” can vary, operationally, functional use of the spasticity-affected upper limb has been defined by Ashford and Turner-Stokes [4]. That is: active task performance; the affected limb actively completes the task or passive task performance; the task is completed by the affected limb with assistance from the unaffected limb or the task is assisted with or completed by a carer; a key area for spasticity interventions [4]. This three-part operational definition of upper limb function is used in the present study.

Multi-disciplinary person-centred approaches are needed to address rehabilitation needs at impairment, activity and participation levels [5–7]. Neuro-rehabilitation clinical practice guidelines recommend collaborative goal setting [7–10], so that patient preferences and priorities can inform programs. Practice guidelines also recommend the use of standardised assessments to measure impairment, activity and participation dimensions of performance relevant to everyday real life [4, 6, 10, 11]. Although most rehabilitation clinicians measure treatment outcomes [12], evidence suggests that many have limited awareness of the range of assessments available [13]. Those who do use assessment use predominantly impairment-based measures—few use measures that capture activity or participation performance [6, 14]. There are measures available. Although an earlier systematic review of functional outcome measures in the hemiparetic upper limb was conducted in 2008 [15], this study was unable to identify a single valid and reliable outcome measure that captured “real life” function. But a more recent review by Ashford and Turner-Stokes in 2013 [4] identified *n* = 27 functional assessments used in upper limb neuro-rehabilitation for people with spasticity (with and without botulinum toxin-A injection). Their inclusion criteria required the assessments to explore function in the context of everyday real life. As yet, these assessments have not been appraised in relation to the psychometric rigour or clinical utility [4, 16]. This study aims to fill that gap.

This study will use the same *n* = 27 assessments from the Ashford and Turner-Stokes study [4] to investigate the psychometric properties of each and draw conclusions regarding their relative rigour and relevance. A key focus will be the validity of these assessments in their ability to capture change in activity performance and life participation. The ICF will be used as the framework to appraise assessment content to determine the extent to which items address activity and participation domains in addition to impairment (body structures and function). Determining the content validity of items in relation to these domains is important not only to see how valid the assessment is in measuring “health” as it is defined by the ICF but also because these domains reflect common patient goals.

Common neuro-rehabilitation goals for people with upper limb spasticity include reducing pain, increasing the range of movement, preventing contractures and reducing spasticity to enable movement training, splinting or casting [6, 12, 17]. Other goals relate to increasing a person’s ability to perform activities and participate in their life situation [6, 11, 16, 18–20]. To date, no systematic review has done this.

Outcomes of this review will be helpful to clinicians and researchers alike working with people who have upper limb spasticity. Right now, some attention has been given to neuro-rehabilitation for people with upper limb spasticity on function in everyday real life [11, 16], but it is a relatively new focus of program evaluation [4, 10, 11] and a challenging one [17, 20, 21]. Determining whether or not interventions impact functional outcomes in everyday life for people with upper limb spasticity has, to date, been complicated by methodological problems, not just in relation to function but also spasticity measurement [15, 22] and the use of weak study designs [17, 21]. There is a need for more research to show that multidisciplinary upper limb spasticity management rehabilitation programs have an impact on the ability of people to perform activities in everyday real life [11, 23, 24].

The objectives of this systematic review are to locate and appraise existing evidence of the psychometric properties of the outcome measures identified by Ashford and Turner-Stokes [4] and classify those measures to conclude the best tool available for the purpose of measuring activity performance and participation outcomes following upper limb spasticity rehabilitation.

## Methods/Design

This systematic review builds on the systematic search conducted by Ashford and Turner-Stokes [4] by synthesising and appraising the research of the psychometric (measurement) properties of outcome measures reported within the published paper. From the 22 studies located in the published search [4], *n* = 33 assessment approaches were identified. On review of those assessment approaches, some were in fact developed for that particular study, for example, three functional tasks (palm hygiene, cutting the fingernails, placing an arm through the sleeve), and consequently do not have published psychometric properties and were excluded from the current study. The remaining *n* = 27 outcome measures had published research investigating their psychometric (measurement) properties and will therefore form the sample for the present study. The authors acknowledge the creation of a degree of assessment selection bias due to this method.

The aims of this systematic review will be:

1. To classify the functional outcome measures reported by Ashford and Turner-Stokes [4] according to whether activity and or participation outcomes following upper limb spasticity rehabilitation are being assessed; activity performance and participation will be defined according to the ICF model [3]; and
2. To locate all of the existing evidence of the properties of the outcome measures, to evaluate the strength of this evidence and to come to a conclusion about the best measure available for the particular purpose of measuring activity and/or participation outcomes following upper limb spasticity rehabilitation.

### Publication/study inclusion criteria

1. The aim of the study should be to develop or evaluate the measurement properties of a measurement instrument identified in the review published by Ashford and Turner-Stokes [4];
2. The instrument should aim to measure activity performance or participation, as defined by the ICF [3]
  Activity performance is defined as “the execution of a task or action by an individual” or requires assistance from or be completed by a carer for the individual. Participation is defined as “involvement in a life situation.”
3. The instrument is evaluated in adult patients, over 18 years of age, with upper limb spasticity (as defined by the authors of the included studies) or patients before or after botulinum toxin injection engaging in upper limb rehabilitation programs (with or without the inclusion of botulinum toxin therapy). A rehabilitation program is one that is devised and implemented by a clinician to work towards achievement of identified goals. Participants can be engaging in the rehabilitation program whilst a hospital inpatient, transitioning to home or be community dwelling.
4. All research studies must be original research, and both conducted and published studies in English within peer-reviewed literature will be considered for this review.

### Publication/study exclusion criteria

This review is concerned with outcomes of upper limb spasticity rehabilitation that identify changes in the performance of an activity or participation as defined by the ICF [3]. Studies that measure activity performance and participation will be included. Studies that measure upper limb spasticity rehabilitation outcomes through assessment of upper limb impairments only, including pain, range of movement, contracture and changes in tone, will be excluded. Outcomes that have been modified in any manner or implemented in a language other than English will be excluded.

### Search methods for the identification of studies

A search will be conducted in Medical Literature Analysis and Retrieval System Online (MEDLINE), Cumulative Index to Nursing and Allied Health Literature (CINAHL) and Excerpta Medica dataBASE (EMBASE). In MEDLINE, a validated search filter for finding studies on measurement properties will be used [25]; we report the planned MEDLINE search strategy in Additional file 1. CINAHL and EMBASE search strategies are available from the authors on request. Searches with the names of each included instruments (in the title) in combination with the terms for the study population as described in the search strategy [see Additional file 1] will be conducted until each instrument has been searched.

### Screening

Once all searches have been exhausted, the abstracts will be downloaded into the reference management system EndNote and duplicates deleted. A study deemed as a duplicate will have authors, setting and location, outcome measures implemented, date and duration of study in common.

The eligibility criteria will first be applied to the title and abstract, and if deemed relevant, the full manuscript will be retrieved to determine eligibility of potential studies. The initial screen and selection will be completed independently by the first author with the second author blindly screening a 10 % selection of articles for eligibility. Debate on the inclusion or exclusion of studies will be resolved by an independent third reviewer and discussion between all three reviewers to reach consensus.

### Data management

Details on studies that were initially selected based on title and abstract, full-text articles that were retrieved and articles included in the review will be documented. Reasons for the exclusion of retrieved full-text articles, particularly in the case of doubtful articles, will also be recorded.

### Data extraction

Data will be extracted from selected studies by the first author utilising a standardised data extraction form. This form will record information related to participants, study design, description of botulinum toxin therapy and rehabilitation program (s), outcome measures administered and their classification according to the ICF (activity performance and or participation focus), psychometric properties, study inclusion/exclusion criteria if available and a brief summary of the findings. The second author will crosscheck all COSMIN ratings.

### Risk of bias assessment

Studies evaluating the measurement properties of an assessment require high methodological quality with a low risk bias to guarantee that appropriate conclusions are drawn about the properties of the measure [25]. Thus, it is important to evaluate those methodological qualities [26]. This review will apply the COnsensus-Based Standards for the Selection of Health Status Measurement INstruments (COSMIN) checklist with 4-point scale version [27]. This version is recommended by the COSMIN developers for use in systematic reviews of measurement properties. The checklist will be applied to assess the quality of the papers reporting on the psychometric properties of the 27 outcome measures, evaluating whether each study meets the standards for methodological quality with regard to internal consistency, reliability (test-retest, inter-rater and intra-rater reliability), measurement error, content validity (including face validity), structural validity, hypothesis testing, cross-cultural validity, criterion validity, responsiveness, interpretability and generalisability [27]. The 4-point scale will allow a methodological quality rating of either “excellent”, “good”, “fair” or ”poor” to be assigned to the study [27]. The COSMIN checklist was developed in an international Delphi study with the focus of evaluating the methodological quality of studies on measurement properties [27]. The COSMIN checklist is a modular tool, and the measurement properties evaluated in the study will determine which components or “boxes” need to be completed [27].

### Data analysis

Individual assessment items within the outcome measures will be examined to extract meaningful concepts. Those concepts will then be linked to the ICF framework categories of activity performance and or participation following the linking rules suggested by Cieza et al. [28] [see Additional file 2]. This linking process will enable the extent to which outcomes are valid measures of activity performance and life participation to be determined.

The COSMIN checklist with 4-point scale version [27] as described above will be applied to the selected studies, as per COSMIN guidelines, to appraise the overall methodological quality of studies. From here, Terwee’s quality criteria for measurement properties [29] will be applied. Quality criteria for the following nine measurement properties are defined; content validity, internal consistency, criterion validity, construct validity, reproducibility, reliability, responsiveness, floor and ceiling effects and interpretability. This data analysis process will enable conclusions to be drawn regarding the strongest psychometric measure available for the particular purpose of evaluating activity and/or participation outcomes following upper limb rehabilitation. Differences in the psychometric properties of outcome measures for patients with and without upper limb spasticity will be discussed.

## Discussion

This systematic review will provide a comprehensive evaluation of the measurement properties of outcome measures assessing activity performance and participation goals for adults with upper limb spasticity undergoing rehabilitation. The results of this review will provide health professionals with detailed information to guide clinical decision-making when choosing the most appropriate outcome measure for occupational performance. Rehabilitation clinicians and managers will also be provided with information to permit accurate assessment and monitoring of the relationship between rehabilitation and health outcome in these patients.

## Additional files

Additional file 1:
**Search strategy.** MEDLINE search strategy. Additional file 2:
**ICF linking rules v2.** Description of how to link concepts to ICF as per linking rules.

## Abbreviations

CINAHL: Cumulative Index to Nursing and Allied Health Literature
COSMIN: Consensus-based Standards for the Selection of Health Measurement Instruments
EMBASE: Excerpta Medica dataBASE
ICF: International Classification of Functioning, Disability and Health
MEDLINE: Medical Literature Analysis and Retrieval System Online
