# Supplementary material for: A systematic review protocol to evaluate the psychometric properties of measures of function within adult neuro-rehabilitation
Source: Syst Rev. 2015 Jun 13;4:86. doi: 10.1186/s13643-015-0076-5 (PMC4470035; doi:10.1186/s13643-015-0076-5)
Supplement: Additional file 2: — ICF linking rules v2. Description of how to link concepts to ICF as per linking rules. [file 13643_2015_76_MOESM2_ESM.pdf]

Additional file 2: ICF Linking Rules according to Cieza et al [26].

Step 1: Application of the specific rules as detailed below.

Specific rules for the linking of health-status measures;

1. Identify all meaningful concepts within each item of the health status measure under consideration
2. The response items of an item are linked if they contain meaningful concepts
3. The interval of time to which the item refers such as "during the last week" is not linked to the ICF
4. If a meaningful concept of an item is explained by examples, both the concept and the examples are linked. However, the ICF category to which the examples have been linked will be put within parentheses. Examples are usually introduced with "e.g." "for example", or "such as" or appear in parentheses.

Specific rules for technical and clinical measures

1. Define the aim with which the corresponding technical or clinical measure is used in form of a meaningful concept. Please consider that the aims can vary from investigation to investigation.

Step 2: Application of the following rules

1. Before one links meaningful concepts to the ICF categories, one should have acquired good knowledge of the conceptual and taxonomical fundaments of the ICF, as well as of the chapters, domains, and categories of the detailed classification, including definitions.

2. Each meaningful concept is linked to the most precise category.

3. Do not use the so-called "other specified" ICF categories, which are uniquely identified by the final code 8. If the content of a meaningful concept is not explicitly named in the corresponding ICF category, the additional information not explicitly named in the ICF is documented.

4. Do not use the so-called "unspecified" ICF categories, which are uniquely identified by the final code 9 but the lower level category.

5. If the information provided by the meaningful concept is not sufficient for making a decision about the most precise ICF category it should be linked to, the meaningful concept is assigned nd (not definable).

Special cases of this rule:

   a. Meaningful concepts referring to health, physical health or mental (emotional) health in general, are assigned nd-gh, nd-ph, or nd-mh (not definable-general health, not definable-physical health, not definable-mental health), respectively.

   b. Meaningful concepts referring to quality of life in general are assigned nd-qol (not definable-quality of life).

6. If the meaningful concept is not contained in the ICF, but it is clearly a personal factor as defined in the ICF, the meaningful concept will be assigned pf (personal factor). Personal factors are defined in the ICF as follows: "The particular background of an individual's life and living, and comprise features of the individual that are not art of a health condition or health states. These factors may include gender, race, age, other health conditions, fitness, lifestyle, habits, upbringing, coping styles, social background, education, profession, past and current experience (past life events and concurrent events), overall behaviour pattern and character style, individual psychological assets and other characteristics, all or any of which may play a role in disability at any level".

7. If the meaningful concept is not contained in the ICF and it is clearly not a personal factor, this meaningful concept is assigned nc (not covered by ICF).

8. If the meaningful concept refers to a diagnosis or a health condition, the meaningful concept will be assigned hc (health condition).
